# Poly-D,L-Lactide-co-Glycolide and Sodium Enoxaparin Composition — an Advanced Coating for Vascular Stents: Biocompatibility and Efficiency Assessment of Stent-Grafts in an Experiment on Large Animals

**DOI:** 10.17691/stm2025.17.6.05

**Published:** 2025-12-29

**Authors:** A.R. Shabaev, N.A. Kochergin, A.Yu. Kanonykina, V.A. Koshelev, A.A. Arnt, A.Yu. Kolesnikov, A.A. Shilov, R.S. Tarasov, Yu.A. Kudryavtseva

**Affiliations:** Junior Researcher, Laboratory of Cellular Technologies, Experimental Medicine Department; Research Institute for Complex Issues of Cardiovascular Diseases, 6 Academician Barbarash Blvd., Kemerovo, 650002, Russia; MD, PhD, Head of the Laboratory of Tissue Engineering and Intravascular Imaging; Research Institute for Complex Issues of Cardiovascular Diseases, 6 Academician Barbarash Blvd., Kemerovo, 650002, Russia; Junior Researcher, Laboratory of Molecular, Translational, and Digital Medicine, Experimental Medicine Department; Research Institute for Complex Issues of Cardiovascular Diseases, 6 Academician Barbarash Blvd., Kemerovo, 650002, Russia; Junior Researcher, Laboratory of Molecular, Translational, and Digital Medicine, Experimental Medicine Department; Research Institute for Complex Issues of Cardiovascular Diseases, 6 Academician Barbarash Blvd., Kemerovo, 650002, Russia; Junior Researcher, Laboratory of Tissue Engineering and Intravascular Imaging; Research Institute for Complex Issues of Cardiovascular Diseases, 6 Academician Barbarash Blvd., Kemerovo, 650002, Russia; Junior Researcher, Laboratory of Tissue Engineering and Intravascular Imaging; Research Institute for Complex Issues of Cardiovascular Diseases, 6 Academician Barbarash Blvd., Kemerovo, 650002, Russia; MD, DSc, Head of the Department of X-ray Surgical Diagnostic and Therapeutic Methods; Kuzbass Clinical Cardiology Dispensary named after Academician L.S. Barbarash, 6 Academician Barbarash Blvd., Kemerovo, 650002, Russia; MD, DSc, Head of the Laboratory of X-ray Endovascular and Reconstructive Cardiovascular Surgery; Research Institute for Complex Issues of Cardiovascular Diseases, 6 Academician Barbarash Blvd., Kemerovo, 650002, Russia; DSc, Leading Researcher, Experimental Medicine Department; Research Institute for Complex Issues of Cardiovascular Diseases, 6 Academician Barbarash Blvd., Kemerovo, 650002, Russia

**Keywords:** vascular stents, stent-graft, arterial perforation, biodegradable polymers, biocompatibility

## Abstract

**The aim of the study** was to evaluate the biocompatibility of vascular stents coated with the membrane based on a copolymer of poly-D,L-lactide-co-glycolide and sodium enoxaparin in an experiment on large animals.

**Materials and Methods:**

A biodegradable coating based on a copolymer of poly-D,L-lactide-co-glycolide (the polylactide-glycolide ratio — 50:50) and low-molecular sodium enoxaparin was applied on 8-mm-long metallic coronary Calipso stents by electrospinning. The sheep carotid artery was implanted with the coated stents (stent-grafts) and uncoated stents. The dynamic patency was assessed by color duplex ultrasound. Three months later, the artery–stent fragments were explanted, fixed with buffered formalin with post-fixation with osmium tetroxide, dehydrated in ethanol and acetone followed by impregnating with epoxy resin. After polymerization, the samples were ground and polished to the required depth. To enhance the contrast after polishing, the samples were treated with Reynolds lead citrate. The samples were visualized by scanning electron microscopy in the backscattered electron mode.

**Results:**

During the three-month experiment, no cases of thrombosis or stenosis of stents and stent-grafts were revealed. A uniform dense neointima up to 165 μm thick formed on the internal surface of the stent-grafts, it was twice as thick as the intima of the intact carotid artery adjacent to the stent-graft. A loose neointima formed on the inner part of the stents without a polymer membrane, reaching 380 μm in some places. All samples demonstrated a classic picture of the formation of a dense fibrous capsule, which separated the metal stent struts from the blood flow and structural elements of the artery, however, the morphology and cellular composition in the samples varied significantly. The struts of the stents without a membrane were surrounded by numerous inflammatory cells. The environment of the stent grafts was represented mainly by smooth muscle cells, fibrocytes, fragments of the elastic membrane located in the intercellular matrix; there were no inflammatory cells. The polymer coating of the stent-grafts completely degraded forming no scar tissue.

**Conclusion:**

The developed polymer coating based on a copolymer of poly-D,L-lactide-co-glycolide (the polylactide-glycolide ratio — 50:50) and the low-molecular sodium enoxaparin for a vascular stent appeared to be effective. When implanted in sheep carotid arteries, the stent-grafts cause no development of thrombosis and stenosis, successfully integrating with the animal artery. In 3 months, complete resorption of the polymer coating occurred with no signs of a chronic inflammatory reaction.

## Introduction

Circulatory diseases still hold leading positions among mortality causes in working-age population, and most of fatal cases (up to 50%) are accounted for coronary artery disease [1–4]. Therefore, timely diagnosis and effective therapy of cardiovascular diseases remain one of priorities in modern medicine.

Coronary blood flow can be restored by both: open surgery and using minimally invasive endovascular procedures. However, in recent decades, X-ray endovascular methods have got a long start over coronary artery bypass grafting for the number of procedures performed; it is due to the less injury rate, a reduced rehabilitation period, and good clinical outcomes of endovascular techniques. Particularly, in the therapy of patients with acute coronary syndrome, the ratio of coronary artery bypass grafting and percutaneous coronary intervention (PCI) is 8–12% and 50–60%, respectively [2].

With advances in technology of X-ray endovascular surgery, there is the ever-growing number of interventions on coronary arteries and other vessels as well, including brachiocephalic, intra- and extracranial arteries, and also the lower limb arteries. Such procedures frequently involve stents of different structures able to provide the blood flow recovery in affected vessels [2]. However, despite significant advances in X-ray endovascular surgery, the problem of complications occurring in both — therapeutic and diagnostic interventions — is still urgent [5–7].

One of the most dangerous complications of PCI is iatrogenic perforation of coronary arteries, its rate, according to clinical studies, is 0.2–0.6% of all interventions [6]. The risk of such complications increases in proportion to the complexity of the performed procedure: in routine PCI their rate is about 0.43%, while in the procedures aimed at recanalization of chronic occlusions of coronary arteries the rate increases up to 2.9% [7]. Those patients who have marked vascular tortuosity and calcified plaques run the most risk to develop perforation that makes the procedure significantly difficult.

The perforation of coronary arteries carries high risk of life-threatening complications including cardiac tamponade, massive hemorrhage, and cardiogenic shock. The fatality rate of these complications reaches 10.7% [5] that governs the relevance of searching for novel preventive techniques and therapy of iatrogenic perforations, as well as the improvement of endovascular technologies aimed at reducing the risk of such complications.

The optimal solution to arrest the bleeding of poorly accessible arteries is to use stent-grafts, i.e., the stents with the outer coating, which covers the artery dissection/ perforation recovering the blood flow. Currently, there are commercially available stent-grafts with the coating from different materials: undegradable polymers (e.g., polytetrafluorethylene, polyurethane) and biological tissues (e.g., hoarse pericardium) [8–10]. Moreover, undegradable polymers, like biomaterials, remain in the arterial wall for a long time and can cause chronic inflammation, and as a consequence, not infrequently can result in calcification in the implantation zone [11, 12].

Biodegradable polymers, especially those with a short resorption period, can serve more effective alternative, since they degrade after performing their protective function in closing perforation and when remodeling the perforated artery, causing no chronic inflammation. Additionally, pharmaceuticals can be included into polymer coating according to the production of drug-coated stents: the pharmaceutical substance is released by doses, so far as the polymer composition is degrading, having a local therapeutical effect.

Previously [13, 14], we screened different polymers with the purpose of creating a stent-graft in relation to elastic and stress-strain properties, bio- and hemocompatibility. The copolymer of poly-D,L-lactide-co-glycolide, the polylactide-glycolide ratio being 50:50, was chosen as the most appropriate and satisfying all requirements: the coating integrity in balloon inflation, high bio- and hemocompatibility, and a short biodegradation period — not exceeding 3 months. Electrospinning was used to apply the coating that enabled to uniformly apply the coating of required thickness, include pharmaceuticals into microfiber composition promoting their releasing by doses after implantation, with the polymer thread destructing. Low-molecular heparin, enoxaparin, was chosen as the pharmaceutical, since in addition to anti-thrombotic and anticoagulant characteristics; it also has an anti-inflammatory effect [15].

For *in vivo* clinical efficiency assessment of the chosen coating, we made a decision to implant with stent-grafts in the large animal artery followed by the risk assessment of thromboses, restenosis, and the response of the surrounding tissues to the polymer coating.

**The aim of the study** was to evaluate the biocompatibility of vascular stents coated with the membrane based on the copolymer of poly-D,L-lactide-co-glycolide and sodium enoxaparin in an experiment on large animals.

## Materials and Methods

**Figure 1.**
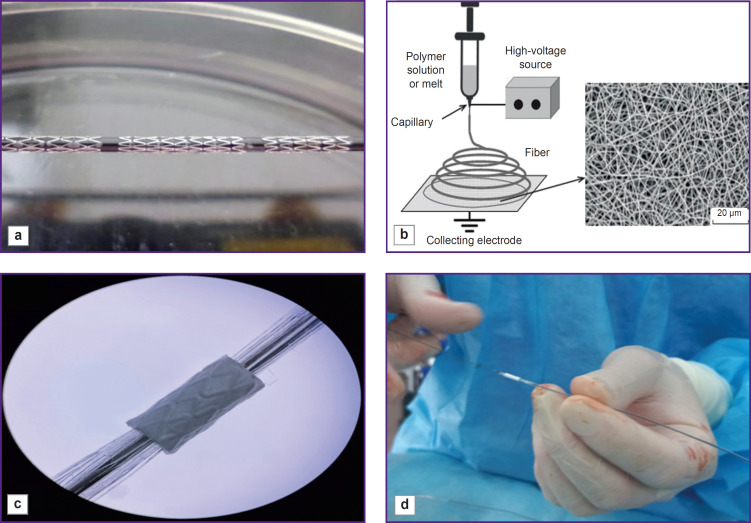
Preparation of stent-grafts for implantation: (a) positioning of stents on a metal shaft; (b) coating application using electrospinning; (c) quality assessment of the applied coating using a stereomicroscope; (d) stent-graft fixation on a balloon

Metallic coronary Calipso stents (Angioline, Russia), 8 mm long, 4 and 4.5 mm in diameter, were used for being coated with a biopolymer membrane. To obtain the coating we prepared 15% solution of the copolymer of poly-D,L-lactide-co-glycolide, the molecular mass being 30,000–45,000 g/mol, and the lactide-glycolide ratio — 50:50 (Novokhim, Russia) dissolved in hexafluoropropanol (1,1,1,3,3,3-hexafluoro-2-propanol), purity ≥99.8% (Sigma-Aldrich, USA). The polymer was mixed up using a magnetic stirrer for 12 h, at room temperature until completely dissolved. To provide hemocompatibility and reduce the risk of thrombosis, we added low-molecular heparin — Enixum (sodium enoxaparin) followed by stirring the solution within 30 min. The stents were positioned on a metallic shaft 1.0 m in diameter (Figure 1 (a)). The polymer coating was applied by electrospinning (Figure 1 (b)) using Nanon-01A (MECC CO, Japan). The quality of the applied coating was assessed using a stereomicroscope (Figure 1 (c)). The polymer coating thickness was 80–90 μm. After drying, the stent-graft was placed on a balloon (Figure 1 (d)) and sterilized by radiation.

The main study stages:

1. a stent-graft implanted into the internal carotid artery of a laboratory animal with the prior assessment of the native vessel condition using color duplex scanning-guided (CDS) intravascular ultrasound (IVUS);
2. follow-up using CDS, the control points — day 3, month 1, and month 3;
3. implantation of stent-grafts in 3 months;
4. histological examination of explants.

The research studies were carried out in accordance with Guide for the Care and Use of Laboratory Animals (National Research Council, 2011), the principles of European Convention for the Protection of Vertebrate Animals used for Experimental and other Scientific Purposes (Strasburg, 2006), World Medical Association Declaration of Helsinki (2024), and were approved by the local ethics committee of Research Institute for Complex Issues of Cardiovascular Diseases (protocol No.11 dated November 20, 2023).

As to prevent the early thrombosis of a stent-graft, prior to the surgery, on the previous evening, the laboratory animal was given the loading dose of clopidogrel, 300 mg, and acetylsalicylic acid, 300 mg. The surgeries were performed under endotracheal anesthesia. The polymer membrane coated stent-grafts were implanted into the animal interior carotid artery on either side. The experiment involved sheep (n=5) weighing 32–35 kg. There were used 7 stent-grafts (an experimental group) and 3 uncoated stents (a control group). Unequal number of stents in the groups was due to the fact that uncoated stent implantation findings were described in the study [16]. Moreover, we confirmed in the experimental study [17] the existing available works [16]. The implantation of the stents with developed biopolymer coating envisaged a thorough study that required increasing the number of observations.

**Figure 2.**
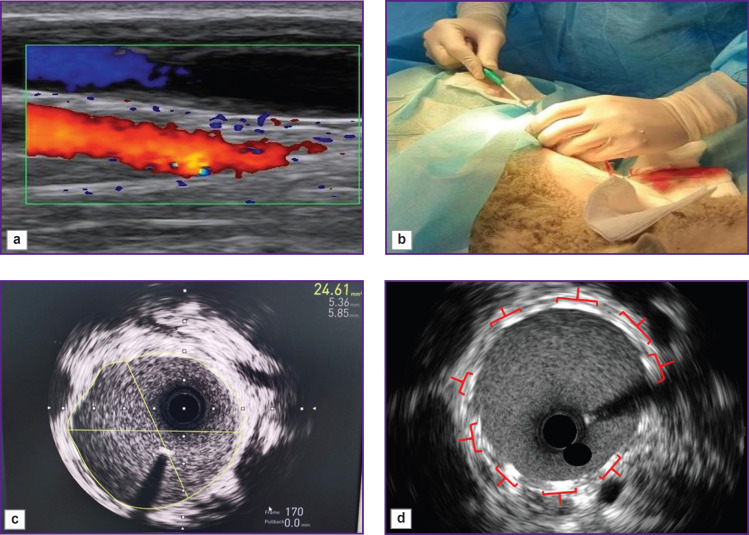
The process of the carotid artery being implanted with a stent-graft: (a) ultrasound imaging of the common carotid artery; (b) sheath positioning; (c) assessment of a vessel reference diameter using an intravascular ultrasound sensor; (d) the stent-graft deployment quality control using intravascular ultrasound

The common carotid artery was implanted with a stent-graft in the following way. After an experimental animal was anesthetized, the common carotid artery was imaged using color duplex US, Mindray M5 (Mindray, China) (Figure 2 (a)) followed by the artery punction and the sheath 6 Fr (Figure 2 (b)) positioning. After systemic administration of heparin (2500 U) we used IVUS system iLab Polaris with OptiCross sensors (Boston Scientific, USA) to assess the vessel reference diameter and choose the necessary size of a stent or a graft (Figure 2 (c)). The artery diameter measured using two different techniques showed the essential difference: IVUS measurement revealed the artery diameter to be on average by 0.8–1.0 mm larger than the preliminary measurements using US. The stent-graft diameter was chosen based on IVUS findings.

Then a stent-graft was positioned parallel to IVUS sensor, 0.5–1.0 cm distally. After positioning, under the guidance of subcutaneous and intravascular US, the common carotid artery was implanted with the stent-graft. The deployment was controlled by IVUS; in case of malposition and/or incomplete deployment, there was performed post-dilation using a balloon (up to 16 atm) followed by the result re-assessment. IVUS was used to evaluate the deployment area, apposition, as well as the presence of dissections (Figure 2 (d)).

After the stent-graft implantation, the balloon and the sheath were removed, the hole was sealed using the suture retention device Angio-Seal (Terumo Medical Corporation, USA).

Intraoperatively, the antibiotic (ceftriaxone, 750 mg, a single dose) was administered to all animals. Postoperatively, the animals had the following therapy: cefuroxime injections (750 g b.i.d. for 5 days after the surgery); ketoprofen 50 mg/ml (2 ml intramuscularly, b.i.d. for 5 days after the surgery); dual anti-platelet therapy up to 90 days after the surgery: clopidogrel (75 mg, orally) and acetylsalicylic acid (75 mg, orally).

The stent functionality was assessed by CDS on day 3, as well as in 1 and 3 months after the surgery. The animals were sacrificed 3 months after the stent implantation. For comparison, an intact (native) carotid arterial fragment adjacent to the stent-graft was chosen as a reference. The explanted stent-grafts/stents were subjected to macro- and microscopic examination. There were assessed the integration of the stents with the carotid artery, the presence of an inflammatory response, the polymer coating degradation pattern, the neointima formation characteristics using an original method: EM-BSEM consisting in embedding the whole fragments of the explanted material into epoxy resin followed by studying using scanning electron microscopy (SEM) in back-scattered electrons [17]. The method enabled to take high-resolution photomicrographies, which were visually similar to the images by transmission electron microscopy.

For this purpose, the explanted “stent-carotid artery” complexes and the intact arterial fragment adjacent to the implanted stent-graft were washed in cooled physiological saline and placed into buffered (pH 7.4) 10% formaldehyde solution (B06-003; BioVitrum, Russia) for 24 h with a single formalin change in first 12 h. At the next stage the samples were post-fixed in 1% osmium tetroxide (19110; Electron Microscopy Sciences, USA) prepared on 0.1 М phosphate buffer, for 12 h, and then stained with 2% aqueous solution of osmium tetroxide for 48 h. After that the samples were dehydrated in ethanol of an increasing concentration (50, 60, 70, 80, 95%) — by two 15-minute changes in each of the mentioned concentrations. Then the samples were stained with 2% alcohol (95% ethanol) uranyl acetate solution (22400-2; Electron Microscopy Sciences, USA) for 5 h, and dehydrated in isopropanol (06-002; BioVitrum, Russia) for 5 h, and in acetone (150495; LenReactiv, Russia) for 1 h followed by impregnating with the mixture of epoxy resin Araldite 502 (13900; Electron Microscopy Sciences) and acetone in the ratio 1:1, for 6 h, pure epoxy resin — for 24 h, and polymerized in fresh epoxy resin in vessels FixiForm (40300085; Struers, Denmark) at 60°C for 24 h. The resulting epoxy modules were ground and polished to the required depth using TegraPol-11 (Struers, Denmark) with the sequential usage of grinding discs, the grain diameter being 9, 6, and 3 μm. After polishing, the samples were contrasted using Reynolds lead citrate (17810; Electron Microscopy Sciences, USA) for 15 min by applying the solution on the polished surface. After washing in bidistilled water, carbon evaporation, 10–15 nm thick, was applied on the epoxy blocks using the vacuum evaporator, EM ACE200 (Leica, Germany).

The imaging of the sample structure was performed using SEM in secondary (backscattered) electron mode on a scanning electron microscope S-3400N (Hitachi, Japan), at accelerating voltage of 10 or 15 kV.

## Results and Discussion

**Figure 3.**
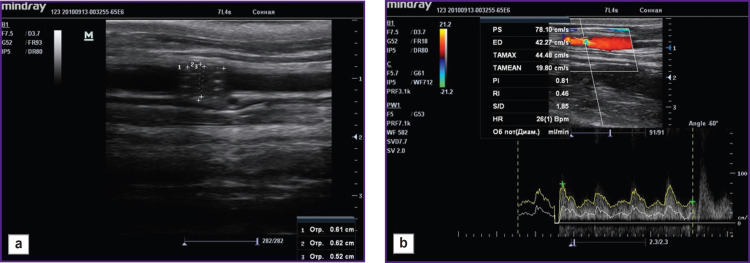
Ultrasound control of the stent-graft patency: (a) imaging of the stent-graft struts; (b) confirmation of the blood flow through a stent-graft

The animals underwent US on day 3, in 1 and 3 months in order to assess the patency of the implanted stents and stent-grafts. The findings confirmed the patency of all implanted stents and stent-grafts: they were well visualized, the struts could be distinctly seen (Figure 3 (a)). The blood flow through the implanted stent-graft was preserved (Figure 3 (b)). No cases of thrombosis were recorded.

After explantation, there was found the deformity of the implanted products: the stents and stent-grafts took the oval shape instead of the expected round shape. It can be due to the fact that the carotid artery was close to the skin, while the coronary stents used in the present study were rather brittle. It made the stent compress and take the oval shape when performing US examination in dynamics due to the sensor placed tightly to the animal skin. It should be noted that the blood flow through the deformed stent was preserved, and no cases of thrombosis of neither stents nor stent-grafts were observed.

**Figure 4.**
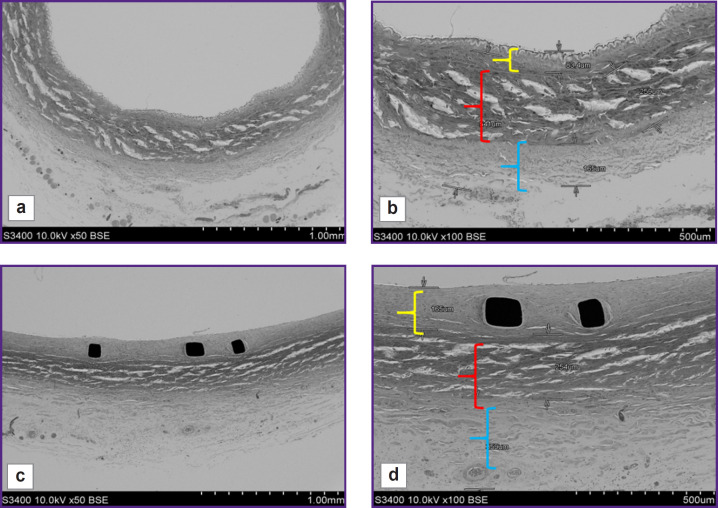
Scanning electron microscopy of the intact artery (a, b) and the explanted stent-graft (c, d) The yellow color indicates intima/neointima zone, red color — media, light blue — adventitia; 50× (a, c), 100× (b, d)

When compared using SEM the condition of the intact artery adjacent to the implanted stent-graft, and the arterial fragment with the implanted stent-graft, we stated that the structure of the arterial layers was similar: there was visualized intima/neointima, the layers of media and adventitia (Figure 4). When hundredfold magnified, it could be seen that the dimensions of media and adventitia layers on average were 250 and 200 μm, respectively, in both — in the intact artery and in the artery with the stent-graft (Figure 4 (b, d)); although the dimensions of the inner layer were different: the intima thickness in the intact artery was no more than 85 μm, while the neointima thickness in the implantation zone was twice as large reaching 165 μm.

Herewith, it should be noted that the neointima was uniform, close-packed, and on the surface there were single endothelial cells. In all samples there were no signs of mural thrombosis. In contrast to the intact artery, the inner elastic membrane in the artery with the stent-graft was not seen due to its compression by the stent in implantation.

**Figure 5.**
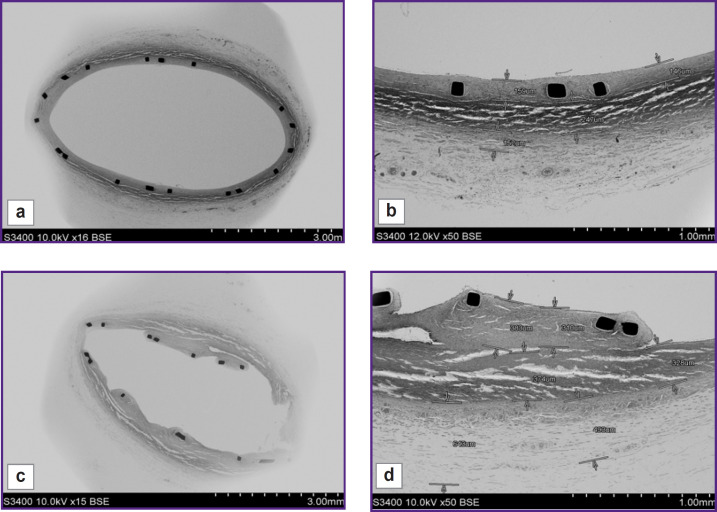
Scanning electron microscopy of the carotid artery fragments including: (a) implanted biopolymer-coated stent-graft; (b) neointima thickness in the stent-graft implantation (*arrows*); (c) uncoated stent; (d) neointima thickness in the implantation of the uncoated stent (*arrows*); (a, c) 15×, (b, d) 50×

Quite another picture could be seen when we compared the artery with the implanted uncoated stents and that with biopolymer membrane coating (Figure 5). In case of the implanted metallic stent without the biopolymer membrane, the neointima was non-uniform, loose, hummocky (Figure 5 (c)), in some places being up to 380 μm thick (Figure 5 (d)).

**Figure 6.**
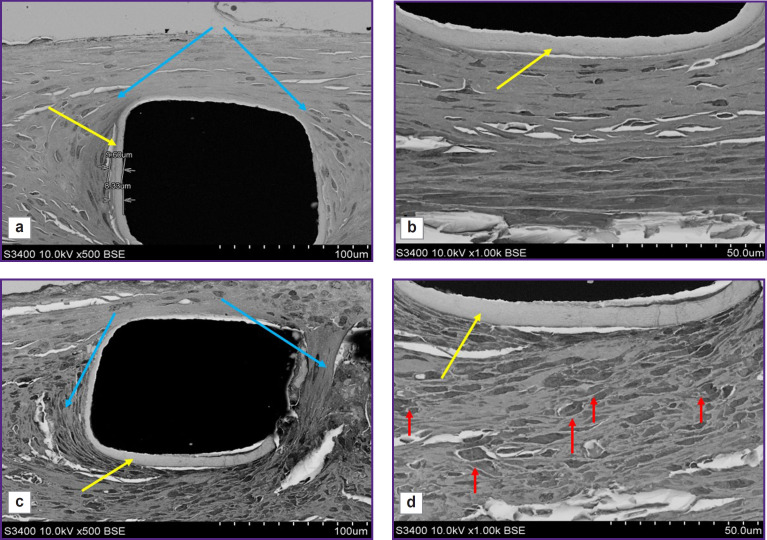
Scanning electron microscopy of the explanted fragments of the stent-grafts with a biopolymer coating (a, b) and the uncoated stent (c, d) The yellow arrows indicate the polymer coating of the stent struts applied by a manufacturer, the red arrows — the inflammatory cells, the pale blue arrows — the fibrous capsule; 500× (a, c), 1000× (b, d)

In 500-fold magnification, there were distinctly visualized the struts of stents and the polymer coating applied by a manufacturer (Figure 6 (a, c)). According to the instruction, to reduce the risk of the neointima growing and rethrombosis, a manufacturer covers the stent struts with a biodegradable polymer — polylactide-co-glycolide with sirolimus added. Such coating was preserved for 3 months after implantation and was easily seen around the struts, its thickness was 8–9 μm.

The arteries with implanted stents and stent-grafts were differed not only in their neointima thickness and morphology, but also in the cellular composition. All samples demonstrated a classic picture of the formation of a dense fibrous capsule, which separated the metal stent struts from the blood flow and the structural arterial elements. 1000-fold magnification (Figure 6 (b, d)) enabled to visualize the remaining polymer coating applied in the stent production; and around there was the newly formed tissue, its composition being significantly different. The struts of the stent without the membrane were surrounded by numerous inflammatory cells (see Figure 6 (d)), while the stent-graft struts were surrounded by a compact layer with no inflammatory signs (see Figure 6 (b)).

**Figure 7.**
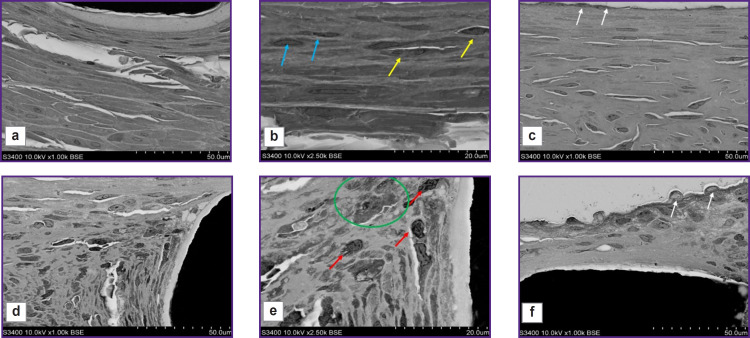
Scanning electron microscopy of the explanted fragments of the stent-grafts with a biopolymer coating (a–c) and the uncoated stent (d–f) The yellow arrows indicate the smooth muscle cells, the pale blue arrows — fibrocytes, the red arrows — macrophages (inflammatory cells), the white arrows — endothelial cells, in the green circle there are giant cells of the foreign body; 1000× (a, c, d, f); 2500× (b, e)

The most representative were the differences surrounded by the struts of the stent and the stent-graft at high-power magnification (Figure 7). The environment of the stent grafts was represented mainly by smooth muscle cells, fibrocytes, fragments of the elastic membrane located in the intercellular matrix; there were no inflammatory cells (Figure 7 (b)). The periphery of the struts of the uncoated stent consisted of macrophages, fibroblasts, and there were single giant cells of the foreign body that was the evidence of chronic inflammation (Figure 7 (d)). At the fibrous capsule border there was the presence of fibroblasts (Figure 7 (e)).

All samples of the stent-grafts 3 months after implantation showed no polymer membrane — a biopolymer coating applied using electrospinning (Figure 7 (a)). It is certain that the polymer coating completely degraded forming no scar tissue.

The surface of the neointima directed towards the blood flow in the samples under study also significantly differed: in the implantation of the stent-graft, on the surface there was an even layer of endothelial cells (Figure 7 (c)), whereas on the surface of the uncoated stent — endothelial like cells, and beneath them there was a loose subendothelial layer represented by fibroblasts and macrophages (Figure 7 (f)).

In the case of implanting the stents without a polymer membrane, there was formed the looser and more thickened neointima. However, the neointima growing into the arterial lumen (towards the blood flow) was moderate compared to the implanted stent-grafts without drug-coating according to the findings of other studies [17]. It is likely to be due to the action of sirolimus included into the composition of the polymer coating of Calipso stents.

There is an opinion that vascular restenosis including the stent implantation zone is related to the proliferation of vascular smooth muscle cells (VSMC), the growth of which can be stimulated by drug-eluting stents [4]. This fact explains the difference in the neointima thickness in the developed stent-grafts and stents with a polymer coating containing sirolimus. The polymer membrane based on poly-D,L-lactide-co-glycolide isolated the metal stent elements from the interior arterial layer preventing the direct contact of the drug coating containing sirolimus with VSMC. In addition to the mechanical separation of the stent structure from the surrounding tissues, there was a positive effect of sodium enoxaparin incorporated into the polymer membrane having a local anticoagulant and anti-inflammatory effect.

Our data appear to be compliant to the findings of the studies [15], which stated sodium enoxaparin as part of composition coatings for stents to decrease the apoptotic rate of endothelial cells and reduce the local inflammatory damage in the implantation in rabbits.

There is one more thing to pay attention to — the difference between the tissues surrounding a stent-graft and an uncoated stent. The latter was found to have fibroblasts — the cells participating in remodeling collagen and elastin fibers in the reparative regeneration process. Fibroblasts contain a great number of lysosomas, which release lysosomal enzymes, and destroying collagen and elastin they promote their rearrangement and the formation of scars in the damaged zone [18]. On the contrary, the tissues surrounding the stents with a biodegradable membrane were found to have only single fibrocytes (the final form of fibroblasts) and VSMC that can indicate the reparative process termination 3 months after the implantation.

Choosing the three-month follow-up period was due to the characteristics of the used copolymer, which exhibits the high rate of hydrolytic degradation due to its low molecular weight [19]. We confirmed the property in both cases: in subcutaneous implantation [14], and also in the present study. At the end of the 3-month period there were found no polymer membrane traces between the metallic stent elements and the arterial wall.

The histological picture also served as the confirmation of a minimal inflammatory response when using the copolymer in the polylactide-glycolide ratio 50:50 compared with other ratios, i.e., with the stents covered by the copolymers with higher polylactide, when the polymer preserved its integrity by the end of the third month [19].

## Conclusion

The experimental study on large animals represented that the developed polymer coating based on a copolymer of poly-D,L-lactide-co-glycolide (the polylactide-glycolide ratio — 50:50) and the low-molecular sodium enoxaparin for a vascular stent appeared to be highly effective. When implanted in the sheep carotid artery, a stent-graft with such coating prevents thrombosis and stenosis contributing to the successful integration with the animal artery causing no chronic inflammation.
